# The Relationship between Single Nucleotide Polymorphisms in Taste Receptor Genes, Taste Function and Dietary Intake in Preschool-Aged Children and Adults in the Guelph Family Health Study

**DOI:** 10.3390/nu10080990

**Published:** 2018-07-29

**Authors:** Elie Chamoun, Nicholas A. Carroll, Lisa M. Duizer, Wenjuan Qi, Zeny Feng, Gerarda Darlington, Alison M. Duncan, Jess Haines, David W.L. Ma, the Guelph Family Health Study

**Affiliations:** 1 Department of Human Health and Nutritional Sciences, University of Guelph, Guelph, ON N1G 2W1, Canada; echamoun@uoguelph.ca (E.C.); ncarro03@uoguelph.ca (N.A.C.); amduncan@uoguelph.ca (A.M.D.); 2 Department of Food Science, University of Guelph, Guelph, ON N1G 2W1, Canada; lduizer@uoguelph.ca; 3 Department of Mathematics and Statistics, University of Guelph, Guelph, ON N1G 2W1, Canada; wqi@uoguelph.ca (W.Q.); zfeng@uoguelph.ca (Z.F.); gdarling@uoguelph.ca (G.D.); 4 Department of Family Relations and Applied Nutrition, University of Guelph, Guelph, ON N1G 2W1 Canada; jhaines@uguelph.ca; 5 University of Guelph, Guelph, ON N1G 2W1, Canada; guelphfamilyhealthstudy@gmail.com

**Keywords:** taste, genetics, diet, health, children, adults

## Abstract

Taste is a fundamental determinant of food selection, and inter-individual variations in taste perception may be important risk factors for poor eating habits and obesity. Characterizing differences in taste perception and their influences on dietary intake may lead to an improved understanding of obesity risk and a potential to develop personalized nutrition recommendations. This study explored associations between 93 single nucleotide polymorphisms (SNPs) in sweet, fat, bitter, salt, sour, and umami taste receptors and psychophysical measures of taste. Forty-four families from the Guelph Family Health Study participated, including 60 children and 65 adults. Saliva was collected for genetic analysis and parents completed a three-day food record for their children. Parents underwent a test for suprathreshold sensitivity (ST) and taste preference (PR) for sweet, fat, salt, umami, and sour as well as a phenylthiocarbamide (PTC) taste status test. Children underwent PR tests and a PTC taste status test. Analysis of SNPs and psychophysical measures of taste yielded 23 significant associations in parents and 11 in children. After adjusting for multiple hypothesis testing, the rs713598 in the *TAS2R38* bitter taste receptor gene and rs236514 in the *KCNJ2* sour taste-associated gene remained significantly associated with PTC ST and sour PR in parents, respectively. In children, rs173135 in *KCNJ2* and rs4790522 in the *TRPV1* salt taste-associated gene remained significantly associated with sour and salt taste PRs, respectively. A multiple trait analysis of PR and nutrient composition of diet in the children revealed that rs9701796 in the *TAS1R2* sweet taste receptor gene was associated with both sweet PR and percent energy from added sugar in the diet. These findings provide evidence that for bitter, sour, salt, and sweet taste, certain genetic variants are associated with taste function and may be implicated in eating patterns. (Support was provided by the Ontario Ministry of Agriculture, Food, and Rural Affairs).

## 1. Introduction

The prevalence of obesity and associated co-morbidities is rising internationally despite ongoing prevention and intervention efforts [1,2]. Therefore, new strategies are warranted to promote the development of effective obesity prevention initiatives. As about half of the risk of developing obesity is heritable [3,4], characterizing the genetic component of obesity and incorporating this information into obesity prevention efforts may be a key part of the complex solution to this global problem. Excess intake of calories due to poor eating habits has been widely recognized as a major factor in the development of obesity, and these habits are established in the earliest years of life [5]. While the genetic basis of these adverse behaviors is not clear, taste preferences have been shown to vary due in part to genetics and to be associated with poor eating habits [6]. Characterizing the genetic factors that predispose to certain taste preferences may therefore provide a tool to tailor eating patterns to promote healthy eating habits. 

The relationship between genetic variation and taste has previously been investigated by examining single nucleotide polymorphisms (SNPs) with outcomes of sensory tests. In particular, studies have focused on the link between taste receptor gene SNPs and measures of taste sensitivity, taste preference, and dietary intake [7,8,9,10,11,12,13,14,15,16]. However, previous studies typically analyze very few SNPs and only measure sensitivity, preference or dietary intake related to one type of taste. In this study, 93 SNPs spanning taste receptor genes that elicit fat, sweet, salt, sour, umami, and bitter tastes were examined for their associations with measures of taste sensitivity and taste preference. SNPs determined to be significantly associated with taste were then examined for potential associations with dietary intake in children. As a result of this comprehensive analysis, SNPs that are associated with taste perception can subsequently be assessed for their effect on the intake of dietary components related to that same type of taste. 

## 2. Methods

### 2.1. Participants

Forty-nine families, including 72 children and 81 adults, were recruited from the Guelph Family Health Study—an existing family-based cohort study. Exclusion criteria included smoking, diagnosis of hypogeusia or ageusia, and having undergone bariatric surgery. Children under the age of 3 years were not recruited due to the potential difficulty in understanding and performing sensory tasks. This study was approved by the Research Ethics Board at the University of Guelph (REB#16-12-629).

### 2.2. Anthropometry, Body Composition, and Blood Pressure Measurements

Parents and their children arrived at the University of Guelph in the Body Composition Lab having fasted for at least two hours. Among both parents, height was measured to the nearest 0.1 cm using a wall-mounted stadiometer (Medical Scales and Measuring Devices; Seca Corp, Ontario, CA, USA) and measured in children to the nearest 0.1 cm using a pediatric length board (Weigh and Measure, LLC; ShorrBoard®, Olney, MD, USA). Body weight was measured while wearing tight-fitting clothing and no shoes using the BOD POD™ digital scale (Cosmed Inc., Concord, CA, USA). Body mass index (kg/m^2^) was calculated from the weight and height measurements. The BOD POD™ was used to determine body composition of adult participants using air displacement plethysmography. Fat mass % in children was determined using bioelectric impedance analysis. Trained research assistants used the Quantum IV – Body Composition AnalyzerTM (RJL Systems, Clinton Township, MI, USA) using single-frequency, with electrodes placed on the right hand and foot. Total body water (TBW) was determined using the Kushner equation [17], then TBW was divided by an age- and sex-specific hydration factor to obtain fat mass %. Among both parents and children, blood pressure and heart rate were measured from the right brachial artery using an automated oscillometric device (HBP-1300 OMRON, Mississauga, Ontario, CA, USA). Cuff size was determined based on arm circumference. Among adults and children, three rested measurements of blood pressure (systolic and diastolic) and heart rate were obtained via an automatic reading while participants were seated in an upright position. The average of the final two measurements for each participant was used in subsequent analyses.

### 2.3. SNP Selection and Genotyping

A PubMed SNP search was conducted for the following genes previously implicated in taste detection: *CD36*, *GPR120*, *GPR40*, *TAS1R1*, *TAS1R2*, *TAS1R3*, *TAS2R38*, *ENaC*, *TRPV1*, *GRM4*, and *KCNJ2*. The resulting SNPs from each gene were filtered by global minor allele frequency (MAF), and SNPs with a minor allele frequency below 5% were removed [18]. The resulting SNPs were filtered using HaploView 4.2 software to obtain tag SNPs (tSNPs). Each tSNP is considered independent due to low linkage disequilibrium (*r*^2^ < 0.05).

Saliva was collected at the health assessment using the Oragene•DNA (OG-575) collection kit for Assisted Collection (DNA Genotek). Participants were fasted for a minimum of 30 minutes before the saliva sample was provided. Genetic material from saliva was extracted by ethanol precipitation according to the manufacturer’s protocol (DNA Genotek). The DNA samples were sent to The Centre for Applied Genomics at The Hospital for Sick Children (Toronto, Canada) where they underwent genotyping using the Agena MassArray System. 

### 2.4. Psychophysical Measurements

Psychophysical tests for adults were administered in sensory booths at the University of Guelph Sensory Laboratory (*n* = 65). Filter paper strips (Indigo Instruments – Cat#33814-Ctl; 47 mm × 6 mm × 0.3 mm) immersed in varying concentrations of tastants were used to determine suprathreshold sensitivity (ST) for the adults only. The tastants were: sucrose for sweet taste (Thermo Fisher Scientific, Rockford, IL, USA; S5-500), monosodium glutamate (MSG) (Thermo Fisher; ICN10180080) and inosine monophosphate (IMP) (Thermo Fisher; AC226260250) for umami taste, sodium chloride (NaCl) for salt taste (Thermo Fisher; S641-500), citric acid for sour taste (A940-500), oleic acid for fat taste (A195-500) (Thermo Fisher Scientific), and PTC for bitter taste (Indigo Instruments, Waterloo, Ontario, Canada,– Cat#33814-PTC). Oleic acid was homogenized in deionized water prior to immersing the filter paper, and all other tastants were dissolved in water at ambient temperature. Filter paper strips were immersed in the tastant solution for about one second before placing them on a drying rack to dry overnight at ambient temperature. This procedure was performed only once for all strips before the study commenced. Taste strips immersed in a solution with the same tastant and concentration were stored together at 4 °C in a small plastic re-sealable bag. Each time a strip was tested, participants placed the taste strip in the middle of their tongue, closed their mouths, and allowed at least five seconds for the tastants to be sensed by taste receptors. Participants were asked to rinse and expectorate with distilled water before beginning and following each strip. Within each taste modality, the range of tastant concentrations tested is shown in Table 1. Oral ST was determined using filter paper strips for a range of tastant concentrations by computing the area-under-the-curve (AUC) of intensity ratings on the general labeled magnitude scale (gLMS) [19], and preference (PR) was measured using a forced-choice paired comparison of hummus samples. Participants were presented with a range of taste strips in random order and were asked to rate the intensity of the strips from 0–100 on a gLMS where 0 = undetectable, 2 = barely detectable, 6 = weak, 18 = moderate, 35 = strong, 52 = very strong, and 100 = strongest imaginable sensation of any kind. For bitter taste, only one rating of PTC intensity was obtained. 

In the PR test for adults, paired hummus samples labeled with random three-digit codes were presented simultaneously to participants in a small translucent sample cup. Each pair of hummus samples consisted of one sample with a standard study formulation and the other with an added ingredient to more strongly elicit a specific taste modality. The standard study hummus was formulated at the University of Guelph Formulation Laboratory. First, chickpeas (540 mL—ARZ Fine Foods) were rinsed in a strainer with cold water and poured into the three-quart polycarbonate bowl of the Robot Coupe Food Processor (Model# R2NCLR). Distilled water (92 mL—President’s Choice), olive + canola oil mix (54 mL—Pur Oliva), lemon juice (10 mL—ReaLemon), tahini (35 mL—ARZ), and salt (5.5 g—Thermo Fisher; S641-500) were then added to the chickpeas. The mix was processed for 40 s, mixed with a spoon to allow chunks of chickpeas on the sides of the processor bowl to be re-incorporated, and processed again for 60 seconds. Five 150 g quantities of hummus were then set aside for the preparation of hummus samples with added ingredients. To elicit stronger fat, salt, sour, sweet, and umami taste, olive + canola oil mix (15 g), salt (0.5 g), lemon juice (7 g), sucrose (4 g), and MSG (4 g) were respectively added to a 150 g quantity of the standard study hummus and mixed thoroughly with a spoon. For each participant, ten sample cups containing five standard hummus samples as well as five hummus samples with added ingredients were prepared (8 g each). A random number generator was used by a research assistant to produce the three-digit codes with which to label the sample cups such that the sensory test administrator was blinded to the hummus formulations. In the PR test, each sample was tasted using a metal spoon following an oral rinse with distilled water. After the second hummus sample was tasted, participants were asked “Which of the two hummus samples did you prefer?” and responded by providing the sensory test administrator with the three-digit code of the preferred sample. Oral ST and PR for all taste modalities were measured during the same study visit.

Children participated in a PR test and a PTC taster test only, following a 2-hour fast (*n* = 60). While the hummus formulations in the PR test were identical to the test with the adults, the forced-choice paired-comparison method was adapted for young children to ensure that the tasks of the procedure would be understood. Once the children provided verbal assent to participate, they joined the test administrator alone in a conference room that was void of any potential distractions. To confirm that the children understood the test, a mock forced-choice paired-comparison task was performed using hair elastic bands of various colors and two containers labeled with a happy face on one and a sad face on the other. The children were asked to choose a “favorite color” and report this color to the test administrator. The children were then presented with two bands, one of which was their favorite color and the other was a different color. The children were then instructed to choose their favorite hair band and place it inside the container labeled with a happy face. If the child placed the hair band with their favorite color into the appropriate container, then they were deemed capable of performing the preference test with the hummus samples. When choosing a preferred hummus sample, the children simply had to point to their preferred sample and the three-digit code of this sample was recorded by the test administrator. Instead of providing an intensity rating on the gLMS for the strip of PTC paper, the children participated in a yes-no task to determine PTC taster status. The children responded with a “yes” or a “no” to the question “Does that taste bad or have no taste at all?” If the children reported a bad taste, they were recorded as “PTC tasters” whereas children who reported no taste were recorded as “non-tasters”.

### 2.5. Dietary Intake of Children

Parents completed a three-day food record for their children, including two weekdays and one weekend day. Parents documented a detailed description of each food or beverage (i.e., cooking method, brand name) and the amount consumed. Food records were inputted into a nutrient analysis program (ESHA Food Processor, Version 11.0.110, Salem, OR, USA). Calories from sugar, added sugar, total carbohydrates, fat, and protein were computed from an average of three days. Energy density of the whole diet as well as the relative contributions of energy density of sugar, added sugar, total carbohydrates, fat, and protein were also computed.

### 2.6. Statistics 

With R Statistical Software Version 3.4.0 (R Foundation for Statistical Computing, Vienna, Austria), generalized estimating equations (GEE) were used first to estimate the regression coefficients for linear models of psychophysical measures of taste and SNPs. Secondly, SNPs significantly associated with a psychophysical measure of taste were then further analyzed using GEE to estimate the regression coefficients for logistic regression models of SNPs and trait pairs including one taste variable and one diet variable. A logistic regression was used as the alleles of each SNP were treated as binomial experiments with *n* = 2 [20,21,22,23]. Only SNPs initially found to be significantly associated with a taste preference in children, prior to the Bonferroni adjustment, were subsequently assessed for associations with dietary intake using logistic regression. Taste variables were generally only paired with diet variables whereby the nutrient elicits that type of taste. For example, SNPs significantly associated with sweet taste preference would only further investigated for associations with added sugar intake. As sour taste is not typically associated with sensing nutrients, it was paired with (1) percent energy from added sugar as sourness often accompanies sweetness in children’s candies, and (2) total energy density of diet to examine any potential global effects of sour taste preference on the diet. GEEs were also used to estimate the regression coefficients for linear models to examine the associations between diet variables and covariates including age, sex, and BMI due to the potential moderating effect of BMI on taste perception [7,19,24,25,26]. Analyses for both parents and children account for correlated outcomes resulting from multiple siblings within some families and from sharing the same household. Regressions were only performed for SNPs located in a gene associated with the same taste modality as the taste outcome. Statistical significance was set to *p* ≤ 0.05. 

## 3. Results

### 3.1. Participant Characteristics

While 72 children and 81 adults from 49 families were recruited for the study, 60 children and 65 adults from 44 families completed the study. Five recruited families did not complete the study due to discontinued communication with the research personnel following recruitment. Adult participant characteristics are summarized in Table 2 and child participant characteristics are summarized in Table 3. Mothers (*n* = 41) and fathers (*n* = 24) had a mean age of 36.3 ± 4.3 years while boys (*n* = 27) and girls (*n* = 33) had a mean age of 4.1 ± 1.2 years. The mean BMI of adults (27.1 ± 5.6 kg/m^2^) indicated overweight and the mean BMI z-score of children (0.30 ± 0.99) indicated normal weight. 

### 3.2. Genetics and Taste Function/Preference

In total, 93 tSNPs were genotyped from thirteen taste-associated genes in both children and adults. Twenty tSNPs were genotyped from fat taste-associated genes (*CD36*, *GPR120*, and *GPR40*), eleven tSNPs were genotyped from sweet taste receptor genes (*TAS1R2* and *TAS1R3*), rs713598 was genotyped from the bitter taste receptor gene *TAS2R38*, twenty tSNPs were genotyped from salt taste-associated genes (*ENaC* and *TRPV1*), twenty-nine tSNPs were genotyped from umami taste receptor genes (*TAS1R1*, *TAS1R3*, and *GRM4*), and twelve tSNPs were genotyped from sour taste-associated genes (*ASIC1* and *KCNJ2*).

As summarized in Table 4, twenty-three tSNPs were associated with a taste outcome in adults before applying a statistical correction for multiple hypotheses. Following a Bonferroni adjustment for multiple hypothesis testing, the rs713598 and rs236514 SNPs remained significantly associated with taste outcomes. The C allele of the rs173598 SNP in the *TAS2R38* bitter taste receptor gene was significantly associated with PTC sensitivity. The A allele of the rs236514 SNP in the *KCNJ2* sour taste-associated gene was significantly associated with sour preference. As summarized in Table 5, eleven tSNPs were associated with a taste outcome in children before applying a statistical correction for multiple hypotheses. Two tSNPs remained significantly associated with a taste outcome in children after applying a Bonferroni adjustment. The C allele of the rs4790522 tSNP in the *TRPV1* salt taste-associated gene was associated with a significantly higher salt preference compared to the A allele in children. The T allele of the rs173135 tSNP in the *KCNJ2* sour taste-associated gene was associated with a significantly higher sour preference compared to the C allele in children. In both parents and children, the C allele of the rs236512 SNP from *KCNJ2* was associated with sour preference. In parents, the A allele of the rs150908 SNP in *TRPV1* was associated with both higher salt taste sensitivity and a lower preference for salt. 

### 3.3. Multiple Trait Analysis: SNPs, Taste and Dietary Intake

Results of the multiple trait analysis are summarized in Table 6. Age, sex, and BMI were not significantly associated with any of the diet variables. The rs9701796 SNP in the *TAS1R2* sweet taste receptor gene was associated with both sweet taste preference (*p* = 0.022) and percent energy from added sugar in the diet (*p* = 0.05). The rs9701796 SNP was also significantly related to sweet taste preference when included in a model with total energy density of diet (*p* = 0.05), however total energy density of diet was not statistically significant in the model. While the rs173135 SNP in the *KCNJ2* sour taste-associated gene was no longer significantly associated with sour taste preference, this SNP was significantly associated with total energy density of diet with sour taste preference included in the model *(p* = 0.03).

## 4. Discussion

This study examined associations between a comprehensive panel of SNPs in taste receptor genes and psychophysical measures of taste across all known taste modalities in both parents and their children. Overall, the findings in this study showed that SNPs in taste receptor genes from all of the different types of taste may contribute to inter-individual differences in psychophysical measures of taste. However, only four SNPs (rs173135, rs236514, rs4790522 and rs713598) were found to be significantly related to a taste outcome after applying a statistical correction for multiple hypothesis testing. 

The rs4790522 SNP, located in the salt taste-associated gene *TRPV1*, was found to be significantly associated with preference for salt in children. Regulation of salt intake, or sodium, is due in part to variation in genes related to homeostatic sodium regulation [27,28,29,30] and to hedonic responses to the taste of salt [8]. Sodium intake is important to monitor due to its role in the development of hypertension, a risk factor for the development of cardiovascular disease [31,32,33,34]. The rs4790522 SNP has previously been shown to change the miRNA binding site of *TRPV1*, suggesting that this SNP may affect the stability of the mRNA precursor to *TRPV1* and prevent translation into its functional protein [35]. The potential decreased functionality of *TRPV1* may reduce salt taste sensitivity and therefore increase the preference of salt in carriers of this SNP. To the authors’ knowledge, no associations have previously been found between the rs4790522 SNP and salt taste. Future studies should also consider examining the rs150908 SNP which exhibited significant associations with both salt sensitivity and salt preference in parents, increasing the potential relevance of this variant for salt taste. Studies with larger sample sizes are warranted to replicate these results in order to better understand the genetic basis for salt sensitivity, and therefore hypertension.

Sour taste is elicited by acidic substances through the depolarization of type III taste bud cells [36]. While sourness is conventionally considered a means to avoid the consumption of spoiled foods, many animals find mildly acidic foods to be palatable. Moreover, genetic factors may be more important than shared environment to determine the pleasantness and intensity of sour taste as 34–50% of the variation in pleasantness and use-frequency of sour foods is attributable to genetics [37]. With the knowledge that there is a genetic basis for the preference for sour foods in humans, Ye et al. (2016) proposed that sour taste is mediated by the potassium ion channel K_IR_2.1, encoded by the *KCNJ2* gene [38]. The rs173135 and rs236514 SNPs in *KCNJ2* were found to be associated with the preference for sour in children and parents, respectively. Moreover, the rs236512 SNP was associated with sour preference in both children and adults. Observing associations with sour preference in two different cohorts suggests that this association may pertain to changes in sour taste function. The genetic basis of human sour taste has not previously been explored through examining *KCNJ2* SNPs. These novel findings provide a foundation for future studies to investigate the genetic basis of sour taste as well as sour food intake. 

Variants in *TAS1R2* and *TAS1R3* sweet taste receptor genes have previously been associated with changes in taste sensitivity to sugar [39,40,41,42,43,44,45,46], the excessive consumption of which is an established risk factor for obesity and chronic disease [47,48,49]. Previous research has implicated SNPs in *TAS1R2* and *TAS1R3* in inter-individual differences in sugar sensitivity [7,10] and dietary intake [6,7,9,11,16]. However, this study is the first to find an association between a SNP in a sweet tasting gene with both sucrose preference and dietary sucrose intake. In an analysis of SNPs together with taste and diet, it was found that the rs9701796 SNP in the sweet taste receptor gene *TAS1R2* was both associated with sweet taste preference and percent energy from added sugar in the children. In a previous study in children and adolescents, rs9701796 was associated with increased waist-height ratio as well as with a higher chocolate powder intake in obese children [14]. In another study of children aged 7–12, rs9701796 was not associated with dental caries, a marker often related to excessive sweet food consumption [50]. More research pertaining to this variant is warranted, particularly to assess its relationship with the consumption of sweet foods. By establishing these types of associations in future studies, genetic loci can be considered risk factors for the overconsumption of sweet foods and be used clinically to indicate the risk of developing obesity and other chronic diseases.

The bitterness of green leafy vegetables including Brassica vegetables is related to the taste of thiol compounds and may be stronger in those homozygous for the C allele at the rs713598 locus in the TAS2R38 taste receptor gene. Non-carriers of the C allele may not taste PTC, and this may then influence the perceived bitterness of Brassica vegetables [51]. While parents in this study exhibited a strong association between rs713598 genotype and PTC tasting, no relationship was observed between rs713598 genotype and PTC taster status in children. Children would be expected to show a stronger genotype-phenotype relationship due to having less exposure to culture at their age; however, the lack of association in this study is likely an indicator of the poor reliability of measuring PTC taste sensitivity in this age group. Children between 3–8 years of age may not have an adequately developed understanding of the quality of bitterness. While the study personnel administered a simple yes-no task to determine PTC taster status in the children, this task may still have been too complex due to the unusual taste and paper format of the stimulus. 

There are some limitations to consider in this study. Firstly, the data obtained by assessing taste sensitivity in parents, using isolated compounds on filter paper strips, cannot be used to make direct associations between genetics and food intake. This can also be considered a strength of the study as the observations made are accurate for specific taste modalities; however, salt taste was not accounted for when MSG taste was analyzed. The use of hummus as a food matrix in this study may have introduced uncertainty due to the perception of texture, temperature, and other matrix-specific qualities; however the use of a food as a stimulus increases the relevance of these results to food preferences and food selection. In addition, participants were tested for sensitivity and preference on only one occasion, but this should be repeated to confirm validity. Medication was not screened prior to the study, and it is possible that medications taken by the participants could have interfered with taste perception. While this study was powered to observe differences in sensory outcomes, the sample size was small and the likelihood of making type II errors would be lower with a larger sample. Finally, the genetic heterogeneity due to the presence of more than one ethnicity in this sample may hinder the interpretation of the results as the minor allele frequencies of SNPs differ depending on the population. However, the statistical methods used in this work account for correlated outcomes as parents share a household and siblings share household and genetics.

## 5. Conclusions

This study demonstrated that SNPs in taste receptor genes may contribute to inter-individual differences in taste sensitivity, taste preference and dietary intake. These findings, based on a comprehensive panel of genetic variants in adults and young children, support the relevance of genetics in explaining variation in taste function. The genetic determinants of taste function are important to understand as they may predispose individuals to developing poor eating patterns. In the future, effective strategies can be developed to improve eating habits and therefore risk of obesity through personalized nutritional recommendations based on unique taste preferences.

## Figures and Tables

**Table 1:** Range of tastant concentrations used for each psychophysical test.

Taste Modality (Stimulus)	Threshold/Suprathreshold (mM)	Preference (mM)
Sweet (sucrose)	2.5–500	6%–36% (*w*/*v* *)
Umami (MSG)	3.13–200	3.13–200
Umami (IMP)	0.313–20	0.313–20
Umami (MSG+IMP)	3.13–200 MSG + 0.5 IMP	3.13–200 MSG + 0.5 IMP
Salt (sodium chloride)	5–100	50–250
Sour (citric acid)	1–15	10–200
Fat (oleic acid)	30–100	50–100
Bitter (PTC)	3 μg/strip	-

Tastants were diluted in distilled water and filter papers were submerged in the solutions. * weight/volume. MSG: monosodium glutamate, IMP: inosine monophosphate, PTC: phenylthiocarbamide.

**Table 2:** Adult participant characteristics in total and separated by sex.

Characteristic	Total	Female	Male
*n*	65	41	24
Age (years)	36.3 (4.3)	35.8 (4.5)	37.2 (4.0)
Systolic Blood Pressure (mmHg)	118.4 (19.1)	113.9 (9.9)	130.1 (12.5)
Diastolic Blood Pressure (mmHg)	72.9 (12.4)	70.3 (8.0)	79.6 (8.8)
Heart rate (beats/min)	69.8 (7.9)	69.9 (9.6)	69.8 (7.9)
BMI (kg/m^2^)	27.1 (5.6)	26.4 (5.6)	28.1 (4.9)
% Body Fat	-	34.3 (8.9)	26.8 (9.0)
Ethnicity (%)			
Caucasian	85	-	-
Other	15	-	-

Means (SD) were computed for all characteristics except for ethnicities, which are presented as percentages.

**Table 3:** Child participant characteristics.

Characteristic	Total
*n*	60
Female	33
Male	27
Age (years)	4.1 (1.2)
Systolic Blood Pressure (mmHg)	102.5 (13.6)
Diastolic Blood Pressure (mmHg)	56.9 (11.2)
Heart rate (beats/min)	91.0 (12.4)
BMI z-score	0.30 (0.99)
% Body Fat	29.3 (6.1)
Ethnicity (%)	
Caucasian	81
Other	19

Means (SD) were computed for all characteristics except for sex and ethnicity, which are presented as frequencies and percentages, respectively. Sample size for each characteristic may vary due to incomplete information from 6 children.

**Table 4:** Associations between single nucleotide polymorphisms (SNPs) in taste receptor genes and suprathreshold sensitivity and taste preference in adults.

SNP ID (Gene)		Taste Modality	Outcome	*p*-Value
rs12137730	(*TAS1R2*)	Sweet	Suprathreshold	0.021
rs2499729	(*GRM4*)	Umami	Suprathreshold	0.031
rs3778045	(*GRM4*)			0.007
rs4908563	(*TAS1R1*)			0.022
rs11759763	(*GRM4*)		Preference	0.007
rs2451328	(*GRM4*)			0.021
rs2451361	(*GRM4*)			0.012
rs2499682	(*GRM4*)			0.020
rs2499729	(*GRM4*)			0.036
rs7772932	(*GRM4*)			0.015
rs937039	(*GRM4*)			0.046
rs9380406	(*GRM4*)			0.007
rs150908	(*TRPV1*)	Salt	Suprathreshold	0.043
rs161386	(*TRPV1*)			0.045
rs222745	(*TRPV1*)			0.036
rs150908	(*TRPV1*)		Preference	0.036
rs2301151	(*GPR40*)	Fat	Suprathreshold	0.016
rs3211816	(*CD36*)			0.014
rs713598	(*TAS2R38*)	Bitter	Suprathreshold	0.003 *
rs236512	(*KCNJ2*)	Sour	Preference	0.041
rs236514	(*KCNJ2*)			0.002 *
rs376184	(*ASIC1*)			0.019
rs643637	(*KCNJ2*)			0.011

Generalized estimating equations were used to estimate the regression coefficients of a linear model including suprathreshold sensitivity and taste preference with SNPs (*n* = 65). Regressions were only performed for SNPs located in a gene associated with the same taste modality as the taste outcome. Following a Bonferroni adjustment for multiple hypothesis testing, the rs713598 and rs236514 SNPs remained significantly associated with phenylthiocarbamide suprathreshold and sour preference, respectively. The Bonferroni adjustment of the reported *p*-values accounted for the number of hypotheses equal to the number of SNPs in genes associated with each taste modality. * *p* ≤ 0.05 following a Bonferroni adjustment for multiple hypotheses.

**Table 5:** Associations between SNPs in taste receptor genes and taste preference in children.

SNP ID (Gene)		Taste Modality	*p*-Value
rs7534618	(*TAS1R2*)	Sweet	0.026
rs9701796	(*TAS1R2*)		0.013
rs4713740	(*mGluR4*)	Umami	0.039
rs4790151	(*TRPV1*)	Salt	0.008
rs4790522	(*TRPV1*)		0.001 *
rs877610	(*TRPV1*)		0.010
rs17108968	(*GPR120*)	Fat	0.029
rs173135	(*KCNJ2*)	Sour	<0.001 *
rs236512	(*KCNJ2*)		0.007
rs236513	(*KCNJ2*)		0.006
rs9890133	(*KCNJ2*)		0.006

Generalized estimating equations were used to estimate the regression coefficients of a linear model including taste preference with SNPs (*n* = 60). Regressions were only performed for SNPs located in a gene associated with the same taste modality as the taste outcome. The rs4790522 (*TAS1R2*) and rs173135 (*KCNJ2*) SNPs remained significant following a Bonferroni adjustment for multiple hypothesis testing. The Bonferroni adjustment of the reported *p*-values accounted for the number of hypotheses equal to the number of SNPs in genes associated with each taste modality. * *p* ≤ 0.05 following a Bonferroni adjustment for multiple hypotheses.

**Table 6:** Multiple trait analysis of SNPs in taste receptor genes, taste preferences and dietary intake in children.

SNP (Gene)	Taste Modality	Dietary Outcome	*p*-Value	
			Taste Preference	Diet
rs17108968 (*GPR120*)	Fat	Total energy density (kcal/g)	0.09	0.46
		Energy from fat (kcal)	0.10	0.69
		% Energy from fat	0.09	0.65
rs4790151 (*TRPV1*)	Salt	Sodium (mg)	0.92	0.30
rs4790522 (*TRPV1*)			0.29	0.44
rs877610 (*TRPV1*)			0.58	0.71
rs173135 (*KCNJ2*)	Sour	Total energy density (kcal/g)	0.20	0.03 *
		% Energy from added sugar	0.39	0.49
rs236512 (*KCNJ2*)		Total energy density (kcal/g)	0.64	0.36
		% Energy from added sugar	0.80	0.35
rs236513 (*KCNJ2*)		Total energy density (kcal/g)	0.34	0.11
		% Energy from added sugar	0.55	0.78
rs9890133 (*KCNJ2*)		Total energy density (kcal/g)	0.34	0.11
		% Energy from added sugar	0.55	0.78
rs7534618 (*TAS1R2*)	Sweet	% Energy from added sugar	0.47	0.11
		Total energy density (kcal/g)	0.32	0.39
rs9701796 (*TAS1R2*)		% Energy from added sugar	0.02 *	0.05 *
		Total energy density (kcal/g)	0.05 *	0.98
rs4713740 (*GRM4*)	Umami	Total energy density (kcal/g)	0.37	0.59
		% Energy from protein	0.37	0.99

Generalized estimating equations were used to estimate the regression coefficients of a logistic model including SNPs with trait pairs including a taste preference variable and a diet variable (*n* = 60). Regressions were only performed for SNPs determined to be significantly associated with taste preferences in the initial linear regressions. Taste variables were generally only paired with specific diet variables whereby the nutrient elicits that type of taste. * *p* ≤ 0.05.
